# Mid-Infrared Lifetime Imaging for Viability Evaluation of Lettuce Seeds Based on Time-Dependent Thermal Decay Characterization

**DOI:** 10.3390/s130302986

**Published:** 2013-03-01

**Authors:** Ghiseok Kim, Geon Hee Kim, Chi-Kook Ahn, Yoonkyu Yoo, Byoung-Kwan Cho

**Affiliations:** 1 Center for Analytical Instrumentation Development, Korea Basic Science Institute, 169-148 Gwahak-ro, Yuseong-gu, 305-806 Daejeon, Korea; E-Mails: ghiseok@kbsi.re.kr (G.K.); kgh@kbsi.re.kr (G.H.K.); 2 Department of Biosystems Machinery Engineering, Chungnam National University, 99 Daehak-ro, Yuseong-gu, 305-764 Daejeon, Korea; E-Mail: zelgadiss00@naver.com; 3 Marine Transportation Research Division, Korea Institute of Ocean Science and Technology, 321312 Beon-gil, Yuseong-daero, Yuseong-gu, 305-343 Daejeon, Korea; E-Mail: yoonkyu.yoo@gmail.com

**Keywords:** mid-infrared thermography, thermal lifetime image, nondestructive test, lettuce seed, seed viability

## Abstract

An infrared lifetime thermal imaging technique for the measurement of lettuce seed viability was evaluated. Thermal emission signals from mid-infrared images of healthy seeds and seeds aged for 24, 48, and 72 h were obtained and reconstructed using regression analysis. The emission signals were fitted with a two-term exponential model that had two amplitudes and two time variables as lifetime parameters. The lifetime thermal decay parameters were significantly different for seeds with different aging times. Single-seed viability was visualized using thermal lifetime images constructed from the calculated lifetime parameter values. The time-dependent thermal signal decay characteristics, along with the decay amplitude and delay time images, can be used to distinguish aged lettuce seeds from normal seeds.

## 1. Introduction

As international competition in agricultural markets has intensified because of the proliferation of free trade agreements between countries, many changes have occurred in the seed industry. In particular, some multinational corporations have essentially monopolized the seed production industry, which accounts for a significant proportion of the agriculture industry. Because increases in food production and improvements in the quality of agricultural products are significantly related to quality control of seeds, extensive research into seed quality evaluation has been undertaken.

Conventional seed viability testing methods, such as the tetrazolium and standard germination tests, are destructive, time-consuming, and labor-intensive. Thus, nondestructive technologies for evaluation of seed viability, including discrimination between bad seeds and healthy seeds, are in high demand among farmers and workers in the seed industry [1]. Near-infrared (NIR) spectroscopy techniques were proposed recently as an alternative to conventional methods of seed viability evaluation. NIR spectroscopy technology has reportedly enabled the classification and separation of viable seeds from non‐viable ones [2,3]. A calibration model for distinguishing viable seeds from nonviable seeds was developed using a partial least squares method, and the Fourier transform NIR (FT‐NIR) technique reportedly has good potential for discriminating between viable and non‐viable lettuce seeds [4]. FT‐NIR spectroscopy technology has also been used to classify normal and artificially aged lettuce seeds [5].

In addition to NIR spectroscopy technologies, new applications for IR thermography of agricultural products and bio‐related materials have been explored because this technique is useful not only for measuring the temperature on the surfaces of objects, but also for detecting subsurface or internal heat intrusions and the heterogeneity of the thermal properties within objects, which could be sensitive indicators of cell viability in living organism [6–8]. It is assumed that IR thermography can be applied to nondestructive examination of agricultural products for defects from diseases, physical damage, and physiological disorders. It was shown that defects in agricultural products manifest as changes in the thermodynamic properties of the affected tissue [9,10]. IR thermography techniques can be used to predict whether a quiescent seed will germinate or die upon water uptake, and they are reported to be able to detect imbibition‐ and germination‐associated biophysical and biochemical changes [11].

Although IR thermography techniques have many merits, such as being nondestructive and non-contact, offering full-field imaging, and providing rapid inspection, they also have limitations, including imprecise depth estimations of thermal signals and environmental sensitivity. These limitations have been overcome by combining several technologies and systems that are supported by computerized image processing techniques. The detection or classification of many defects using IR thermography has increased significantly as a result of these combined technologies. However, regardless of the complexity of the information that can be derived from imaging, techniques for analyzing the relationships between the thermal signal and the physical or chemical properties of the object still require improvement.

In the present study, we used lifetime (*τ*) analysis under thermal excitation to evaluate seed viability. This technique is commonly used to characterize biological objects, especially among those fluorescence imaging techniques that use microscopy. In this technique, the lifetime of an object is its residence time in the excited state. This residence time ranges from a few nanoseconds to milliseconds, depending on the excitation method. Fluorescence lifetime imaging techniques using microscopy have been applied in the study of many biological systems for different purposes and are becoming widespread owing to the commercial availability of advanced CCD cameras and image processing hardware [12–15]. A fast global fitting algorithm and iterative convolution have been used to extract two lifetime components from simulated and measured fluorescence lifetime microscopic imaging. The results showed that the distribution of fluorophores and their biochemical environment are generally correlated with the morphology of cells and tissues [16]. A recently developed time‐resolved multispectral laser‐induced fluorescence imaging technique uses tunable wavelengths in the visible spectrum for sample excitation and is capable of nanosecond‐scale characterization of fluorescence responses (lifetime imaging). Using this technique, a large difference was observed between the lifetime characteristics of the fluorescence responses of intact objects and contaminated objects [17].

In the current study, we constructed an IR thermal system consisting of a mid-IR-range (3–5 µm) camera and halogen lamps. We then analyzed the IR thermal signals from seeds to discriminate between aged seeds and healthy seeds using pulsed thermography. In this method, an individual rectangular heat pulse is generated by the halogen lamps, and the characteristic thermal responses of objects are analyzed using both decay amplitude images and delay time images that are reconstructed using the regression coefficients of a thermal decay model. The characterization of signals and their reconstruction using fitting models are commonly used in visible/NIR spectroscopy as a useful analysis method. This signal reconstruction method was adjusted to overcome the limitations of conventional IR thermography and image processing techniques used in thermography. The regression coefficients of treatment groups were compared using one-way analysis of variance, with a *post hoc* Student-Newman-Keuls (SNK) multiple comparison test.

## 2. Materials and Methods

### 2.1. Sample Materials

**Figure 1.**
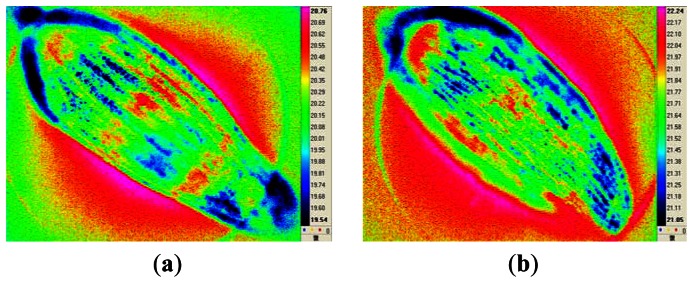

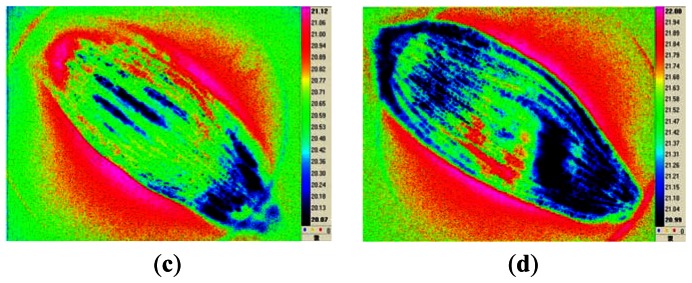
Infrared thermal images of a healthy seed (**a**), and seeds that were artificially aged for 24 h (**b**), 48 h (**c**), and 72 h (**d**).

Lettuce (*Lactuca sativa L.*) seeds, cultivar “Red Leaf”, were acquired from a seed production company. Seed samples were dried to a moisture content of 20%, vacuum packaged, and stored in a water bath (45 °C) for up to 72 h to artificially degrade them [2,5]. Figure 1 shows infrared thermal images of a representative healthy seed and representative seeds that were artificially aged for 24, 48, or 72 h.

**Figure 2.**
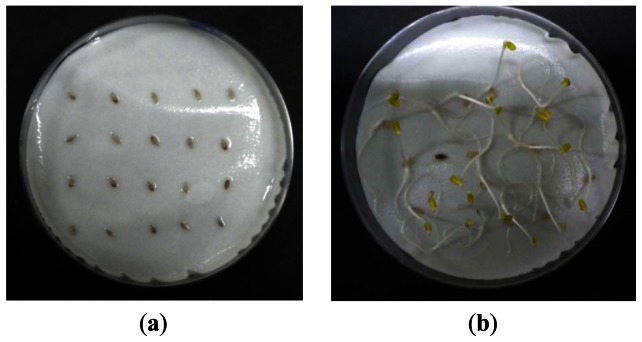
Seed viability test of lettuce seeds before germination (**a**) and after germination (**b**).

Each sample group used in the experiment contained 20 seeds, and all seed samples were dried in an incubator at 20 °C to minimize differences in moisture between samples. The color and shape of all 80 seeds used in the experiment were visually identical. After the IR thermography tests were completed, a standard germination capacity determination (ISTA) was performed for 10 days to determine the viability of all seed samples, as shown in Figure 2.

### 2.2. Infrared Thermography

**Figure 3.**
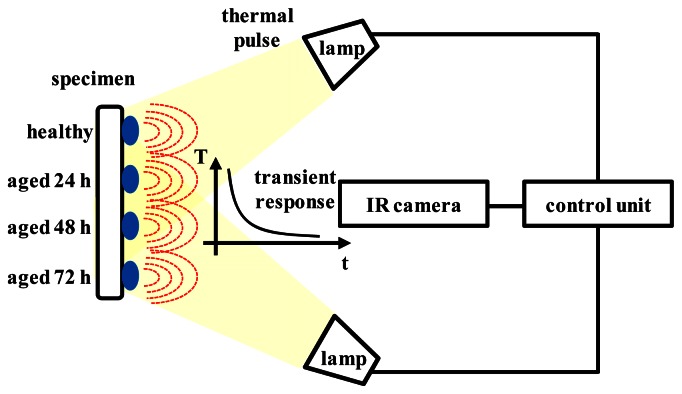
Schematic diagram of pulse thermography system used to obtain images of the thermal responses of lettuce seeds.

Figure 3 shows a schematic diagram of the pulse thermography system used for thermal image measurement of the seed samples. This system consisted of a mid-IR camera, halogen lamps, and a controller. Thermal images of seed samples were taken with the IR camera (SC7600; FLIR Systems, Wilsonville, OR, USA) that had a resolution of 640 × 512 pixels and was sensitive in the 1.5–5 µm spectral range. The detector in the scanner unit was indium antimonide (InSb) and was cooled using an integrated stirling cooler. The system sensitivity was 18 mK at 25 °C. A microscope lens with a 3.2 × 2.6 mm^2^ field of view was used to fit the full length of a seed across the image. Two halogen lamps (100 W each) were operated by a control unit that controlled the pulse on/off time and measurement parameters. The digital image signal was transferred to a computer via a USB or Camlink port. The thermal images were then registered and preliminarily processed using the commercial Altair software (FLIR Systems). This software contains numerous functions for processing thermographic signals and also enables the export of individual images and whole-image sequences in text format. For the post-processing of thermal images, we created thermal image analysis software using MATLAB (ver. 8.0, MathWorks, Natick, MA, USA). The thermal radiation from the seed samples was measured under controlled laboratory conditions at a temperature of 20 °C and relative humidity of 60%. The distance between the halogen lamps and the seed samples was 0.3 m. Thermogram sequences were recorded at a frequency of 5 Hz for 100 s. Each sequence contained 500 images. To analyze the response of the seed samples to the heat pulse, cool-down images were recorded after the halogen lamps were turned off.

Both pulse thermography and thermographical signal reconstruction methods were used to analyze the thermal radiation from seed specimens after a heat pulse. Seed specimens were submitted to a 3 s thermal pulse generated by the halogen lamps. After the thermal wave came into contact with the seed surfaces, it propagated through the specimens. The temperature variation on the surface and in the subsurface was recorded using the IR camera. We statistically processed sequences of these thermal images to estimate the mechanical and physiological factors that are significantly related to seed viability. Temporal noise in the thermal image data, mainly from instruments and the environment, can be suppressed using image processing techniques such as logarithmic time evolution and curve-fitting methods. Additional image processing methods, such as image convolution and background subtraction, were employed to enhance the visibility of the images.

Individual pixel intensity values were normalized to the maximum image intensity. The decay characteristics of thermal radiation from seed samples were expressed by Equation (1), and the regression coefficients (*a*, *b*, *c*, *d*) were obtained using a regression method for the data sets with different aging times. Equation (1) is a general equation describing time-dependent exponential decay, where *y* is the time-dependent thermal radiation; *a* and *c* are the first and second decay amplitudes of thermal decay, respectively; and *b* and *d* are the first and second delay times of thermal decay, respectively. The commercial software MATLAB was used for pixel-based regression analysis of thermal radiation from the seed samples and the reconstruction of lifetime images using the regression coefficients:

(1) $$y=a\exp(\frac{-t}{b})+c\exp(\frac{-t}{b})$$

where *t* is the time on the decay curve. The regression coefficients were obtained using a weighted least square method in which the cost function is given by Equation (2):

(2) $$S=\sum_{i=1}^{n}\omega_{i}{\left(y_{i}-{\hat{y}}_{i}\right)}^{2}$$

where *ω_i_* are the weight parameters, *y_i_* are the calculated values from Equation (1), *yˆ_i_* are the measured values, and *i* represents individual data points.

Analysis of variance with a *post hoc* SNK test for multiple comparisons was performed using the commercial statistics software SPSS (ver. 18, IBM, Armonk, NY, USA) to verify the significance of differences between the regression coefficients of each seed group. The differences between means were assumed to be significant if the *p* value was less than 0.05.

## 3. Results and Discussion

### 3.1. Viability Test

After the IR thermography tests were completed, ISTA was performed for 10 days to determine the viability of seeds in the four treatment groups (a total of 80 seeds) used in the experiments. The germination rate of the healthy seed group was 95%, whereas that of seeds aged for 24 h was <10% after the first 3 days. Seeds aged for 48 and 72 h did not germinate before the end of the viability test.

### 3.2. Time-Dependent Thermal Responses

**Figure 4.**
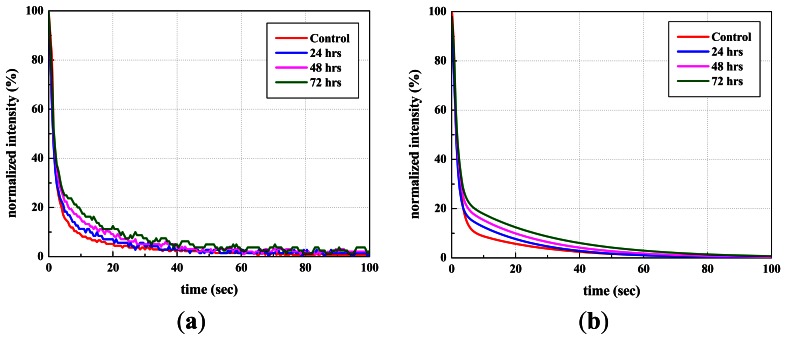
Normalized thermal decay curves of representative lettuce seeds that were not aged (healthy seeds) or aged for 24, 48, and 72 h. Graph (**a**) shows the measured curves, and graph (**b**) shows the curve fitted with (a).

Figure 4 shows the decay characteristics of thermal radiation for a representative healthy seed and representative seeds artificially aged for 24, 48, or 72 h.

**Figure 5.**
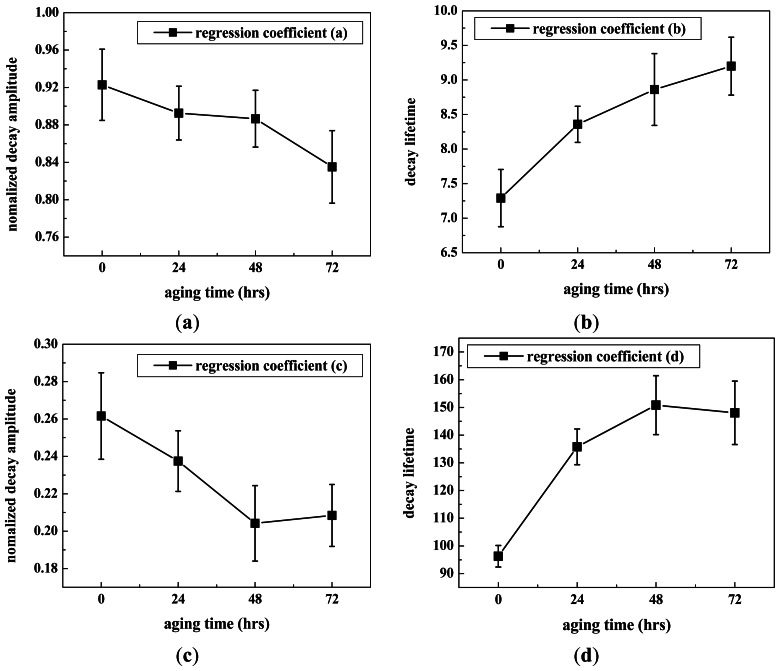
Regression coefficients of thermal decay signals from groups of healthy seeds and seeds aged for 24, 48, or 72 h. Values are represented as mean ± standard deviation.

Table 1 and Figure 5 show the average and standard deviation of the regression coefficients from the decay fit model of each seed group. Differential time-dependent responses were observed for all the regression coefficients. As the aging time of seeds increased, the regression coefficients *a* and *c*, which represent the decay amplitudes, decreased, and the regression coefficients *b* and *d*, which represent the delay time, increased. The thermal radiation of healthy seed samples showed higher decay amplitude and a shorter delay time than those of the aged seed samples. The results of the post hoc SNK multiple comparisons test are shown in Table 1 for the performance evaluation of multiple regression decision rules. Means were considered to be significantly different if the SNK test yielded a *p* value of less than 0.05.

Table 2 shows the variation rates of the regression coefficients for each aging time. The maximum variation rate of 59.18% was observed for coefficient *d*, and the parameters *a*, *b*, and *c* were analyzed as 9.5%, 26.18%, and 15.13%, respectively. This suggests that coefficient *d*, which represents the delay time, can be the most significant variable for determining the viability of seeds using time-dependent thermal decay characterization.

**Figure 6.**
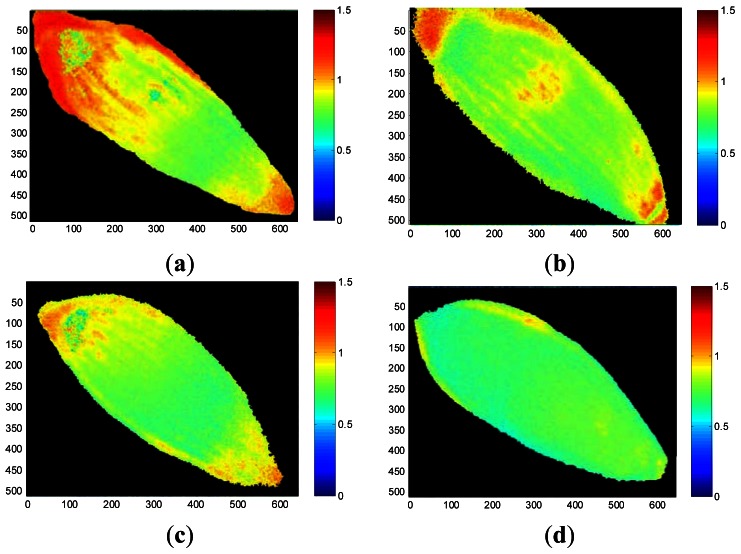
Thermal decay amplitude images of representative healthy seed (**a**) and seeds aged for 24 h (**b**), 48 h (**c**), and 72 h (**d**), produced using an exponential decay model.

**Figure 7.**
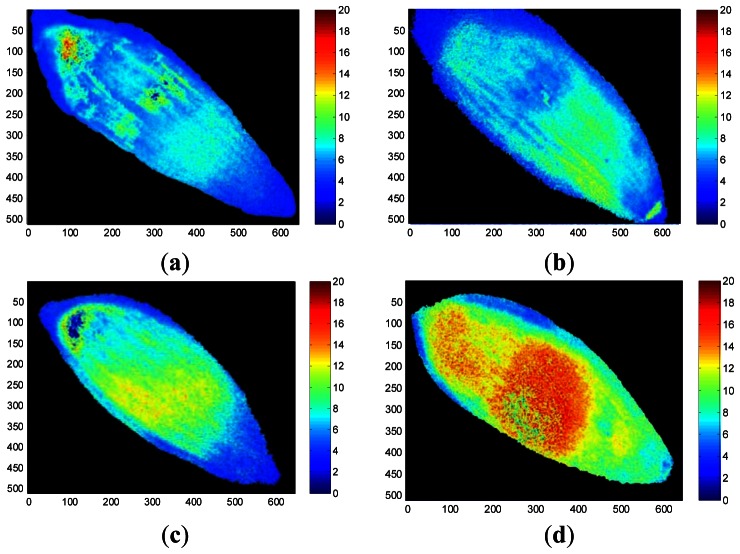
Thermal decay delay-time images of representative healthy seed (**a**) and seeds aged for 24 h (**b**), 48 h (**c**), and 72 h (**d**), produced using an exponential decay model.

False-color thermal decay amplitude images and delay time images of representative seeds from each treatment group are presented in Figures 6 and 7, respectively. The images were reconstructed using the regression coefficients of the pixel-based decay model fit for the entire 500-image sequence acquired for each seed. The aged seed groups generally show lower decay amplitudes and longer delay times than the healthy seed group, regardless of aging time. Reconstructed imaging results obtained from the decay amplitude and delay time show the same trend as the results from the regression coefficients shown in Figure 5. As the aging time increased, the concomitant pixel value in the decay amplitude images that reflect the regression coefficients *a* and *c* decreased, whereas the pixel values in the delay time images reconstructed from regression coefficients *b* and *d* increased.

These results from Figures 4, 5, 6 and 7 showed consistent features. In a highly viable seed group (control seed samples), the decay amplitude of thermal radiation was larger than that of the artificially aged seed groups, and it decreased with increasing aging time. On the other hand, compared with the control seed samples, the artificially aged seeds showed significantly delayed thermal radiation, especially in the delay time parameter *d*. Biophysical conditions such as thermal radiation from seeds can be associated with biological phenomena such as seed aging or cell death accompanied by changed membrane permeability. The membrane is selectively permeable and able to regulate what enters and exits the cell, thus facilitating the transport of materials needed for survival, such as carbon dioxide (CO_2_), oxygen (O_2_), and water. It is known that aged or dead seeds take up water in air more rapidly than healthy seeds [11]. Therefore, thermal radiation measured from artificially aged seed samples can be distinguished from healthy seeds after transient heat excitation. This was demonstrated by the significantly delayed thermal radiation from aged seeds. Accordingly, the decay amplitude or delay time parameters of thermal radiation from seeds, which are associated with water movement across the seed membrane, can be a clue to discrimination between healthy and aged seed samples.

These observations suggest that quantitative methods developed by using differential time-dependent thermal responses such as the regression coefficients of the decay model fit, decay amplitude imaging, and delay time imaging can be used to assess seed viability and as a nondestructive technique for sorting healthy and aged seeds, which are currently inspected using conventional destructive, time-consuming, and labor-intensive seed sorting methods.

## 4. Conclusions

In this study, we demonstrated the utility of an IR thermography method for viability measurements of lettuce seeds, cultivar “Red Leaf”, based on time-dependent thermal decay characterization. The advantages of this method are that it allows the measurement of temperature on the surfaces of objects and the detection of subsurface or internal heat intrusions and the heterogeneity of thermal properties within objects using nondestructive, non-contact, and full-field imaging. We adjusted a pulse thermography method and a thermographic signal reconstruction method by using the regression coefficients of a thermal radiation decay model fit to quantitatively assess the viability of lettuce seed.

The results demonstrated that after the extinction of a heat pulse, the time-dependent thermal signal decay characteristics can depend on the seed aging conditions or seed viability, which are significantly associated with changes in the seed membrane permeability. Decay amplitude images and delay time images were also analyzed to quantitatively distinguish aged lettuce seeds from healthy seeds. To date, most IR thermography has had limited ability to resolve images on a microscopic scale. Moreover, lifetime imaging methods are not commonly used in IR thermography applications until now. The application of a lifetime imaging technique at microscopic scales (FOV: 3.2 × 2.6 mm^2^) using IR thermography has great potential for use in many other scientific areas, including agricultural products and biomaterial research.

## Figures and Tables

**Table 1.** Averaged regression coefficient for lettuce seeds aged for 0, 24, 48, or 72 h.

**Seed Groups**	**Regression Coefficients**			

	***a***	***b***	***c***	***d***
Healthy seed	0.9228 A (0.0762)	7.2907 A (0.8279)	0.2664 A (0.0463)	99.7174 A (7.7712)
Aged 24 h	0.8926 A,B (0.0574)	8.3576 B (0.5225)	0.2419 A,B (0.0325)	137.2989 B (12.9206)
Aged 48 h	0.8866 A,B (0.0606)	8.8619 B,C (1.0405)	0.2273 B (0.0404)	158.6030 C (21.2886)
Aged 72 h	0.8351 B (0.0775)	9.1997 C (0.8376)	0.2261 B (0.0331)	158.7314 C (22.9116)

A,B,C Different letters in each column indicate significant differences (*p* < 0.05). The standard deviation for each mean is provided in parentheses.

**Table 2.** Variation rate of regression coefficients of healthy seed and seeds aged for 24, 48, or 72 h.

**Seed Groups**	**Regression Coefficients**			

	***a***	***b***	***c***	***d***
Healthy seed	0%	0%	0%	0%
Aged 24 h	3.27%	14.63%	9.2%	37.69%
Aged 48 h	3.92%	21.55%	14.68%	59.05%
Aged 72 h	9.5%	26.18%	15.13%	59.18%
